# Prediction Model for Airborne Microorganisms Using Particle Number Concentration as Surrogate Markers in Hospital Environment

**DOI:** 10.3390/ijerph17197237

**Published:** 2020-10-03

**Authors:** Ji Hoon Seo, Hyun Woo Jeon, Joung Sook Choi, Jong-Ryeul Sohn

**Affiliations:** 1 Department of Health & Environmental Science, Korea University, Seoul 02841, Korea; wlgns304@korea.ac.kr; 2 BK21PLUS Program in Embodiment: Health-Society Interaction, Department of Public Health Sciences, Graduate School, Korea University, Seoul 02841, Korea; 3 Department of Health and Safety Convergence Science, Korea University, Seoul 02841, Korea; 2012250541@korea.ac.kr (H.W.J.); pooh03788@korea.ac.kr (J.S.C.)

**Keywords:** indoor air quality, bioaerosol, hospital environment, prediction model, particle number

## Abstract

Indoor microbiological air quality, including airborne bacteria and fungi, is associated with hospital-acquired infections (HAIs) and emerging as an environmental issue in hospital environment. Many studies have been carried out based on culture-based methods to evaluate bioaerosol level. However, conventional biomonitoring requires laborious process and specialists, and cannot provide data quickly. In order to assess the concentration of bioaerosol in real-time, particles were subdivided according to the aerodynamic diameter for surrogate measurement. Particle number concentration (PNC) and meteorological conditions selected by analyzing the correlation with bioaerosol were included in the prediction model, and the forecast accuracy of each model was evaluated by the mean absolute percentage error (MAPE). The prediction model for airborne bacteria demonstrated highly accurate prediction (*R*^2^ = 0.804, MAPE = 8.5%) from PNC1-3, PNC3-5, and PNC5-10 as independent variables. Meanwhile, the fungal prediction model showed reasonable, but weak, prediction results (*R*^2^ = 0.489, MAPE = 42.5%) with PNC3-5, PNC5-10, PNC > 10, and relative humidity. As a result of external verification, even when the model was applied in a similar hospital environment, the bioaerosol concentration could be sufficiently predicted. The prediction model constructed in this study can be used as a pre-assessment method for monitoring microbial contamination in indoor environments.

## 1. Introduction

Interest in indoor air quality is rapidly growing as living standards improve and health awareness increases around the world. Most people spend 80–90 percent of their days indoors. This is especially true in Korea, where Asian dust and general deterioration of air quality are making people more likely to stay indoors. Therefore, indoor air quality is closely related to our health [1]. Indoor air contains both non-biological aerosols and biological aerosols (bioaerosols). Bioaerosols include metabolites from bacteria, fungi, viruses, and endotoxins. These bioaerosols contribute from 5 to 34% of total indoor air pollution, and they are associated with health risks that have various respiratory symptoms, such as decreased pulmonary function, rhinitis, allergic diseases, and asthma [2,3,4,5]. Exposure to airborne microbes may have a more severe effect on people in health-sensitive groups, especially those who are immunocompromised or those who suffer from respiratory and allergic diseases. Altogether, these at-risk groups account for one-third of the world’s population [6].

A hospital is a representative facility that contains sensitive groups of people, along with daycare centers, postpartum care centers, and nursing facilities for the elderly. In Korea, medical facilities with a total area of more than 2000 m^2^ or more than 100 beds are designated and managed as multiuse facilities through the indoor air quality management law (Ministry of Environment). The prevalence of excessive quantities of particulate matter (PM) and total airborne bacteria was found to be high in hospitals [7]. On the other hand, only about 10% of the managed facilities are measured every year because of budgetary and manpower limitations, so blind spots occur in indoor air quality monitoring, even though microbiological monitoring in the hospital environment is important for the prevention of hospital-acquired infections (HAIs) in medical facilities and maintaining a healthy indoor environment.

Indoor air quality in hospitals is emerging as a worldwide hygiene and health problem. Hospital-acquired infections (HAIs) caused by pathogenic and non-pathogenic bacteria are of particular concern worldwide. With the increasing number of patients and people with weakened immune systems due to various diseases, medical facilities are facing ever more problems with HAIs, and HAIs have become an important factor in patient morbidity and mortality. Therefore, prevention of infections from airborne bacteria or fungi is critical in the context of medical facilities. HAIs are also associated with the progress of medical treatment and medical costs from a more macroscopic perspective [8,9].

In general, monitoring of bioaerosols is time-consuming and requires specialized personnel. In addition, it is difficult to accurately represent bioaerosol contamination levels in medical facilities because the concentration at a particular sampling site is valid only for the moment of measurement [8,10]. Previous studies attempted to evaluate the predictability of bioaerosol concentration using real-time particle counting, which is faster and simpler than culture-based measurement of microbes in the air. The results have been inconsistent: while some studies showed a significant correlation between particle number and biological aerosol [2,8,11], others demonstrated no correlation [10,12]. In addition, most studies have focused only on the operating room and intensive care unit (ICU) [9,12,13], and a few studies have investigated other wards of the hospital [8]. Consequently, the question of whether particle counting can predict the concentration of airborne microbes remains controversial. Overall, the predictability of airborne microbes in hospital spaces (including clinics) has rarely been investigated.

In order to provide an analytical basis to address these challenges, this study aimed to (1) comparatively evaluate bioaerosol and particle concentration in various wards of the hospital, and perform correlation and regression analysis of bioaerosol contamination by particle size; (2) construct the prediction model via particle counting methods for the prediction of microbial populations in the hospital environment; and (3) validate the model and evaluate its utility for different hospital environments.

## 2. Materials and Methods

### 2.1. Monitoring Campaign

Monitoring campaigns were carried out in five medical facilities from spring to late summer (6 March–25 September 2019). Seoul, where the study site was located, has the highest population density among the capital cities in Organization for Economic Cooperation and Development (OECD) countries, with a population of 10 million [14]. The characteristics of the surveyed hospital facilities are shown in Table 1. According to the size of the facility, each was classified as either a general hospital or a clinic. Biomonitoring was performed at 30 points in different wards in the hospital, such as the internal medicine, surgical ward, general ward, treatment rooms, patient rooms, and central supply room (CSR), in which equipment and medical instruments are sterilized and stored. Airborne bacteria and fungi were selected for measurement because they are the most representative bioaerosols in the general environment on the Korean Peninsula [15,16]. Particle numbers were also monitored in real-time in order to analyze the correlation between particle numbers and bioaerosol concentration.

### 2.2. Airborne Bacteria and Fungi

An Anderson sampler with 400 (*N*) −0.25 mm holes (MAS-100 Eco; MERCK, Kenilworth, NJ, USA) and an operating pump (Buck, flow rate: 100 L/min; AP Buck Inc., Orlando, FL, USA) was used to measure bacterial and fungal bioaerosols. The sampler has been used in related studies [4,17]. Sampling was conducted three times at intervals of about 15 min in the morning and afternoon at all sampling points. To prevent contamination, the sampler was cleaned with 70% alcohol before and after each sample was taken. All samples were sealed with parafilm after the sample ID was entered, placed in an icebox keeping below 4 °C, and moved to the laboratory [18]. For culture-based analysis, the bacterial particles were plated onto TSA (tryptic soy agar) and incubated at 30 ± 1 °C for 48 h. The potential fungal particles were deposited on MEA (malt extract agar) and incubated at 25 ± 1 °C for 4 ± 1 days. Colonies growing on both media were counted as colony-forming units per cubic meter (CFU/m^3^). During the bioaerosol sampling period, meteorological conditions including temperature (°C) and relative humidity (RH, %) were also monitored and recorded using a portable weather station (Kimo) at each sampling point.

### 2.3. Size-Segregated Particle Counting

The size-segregated particle number concentration was measured using an optical particle counter (Grimm1.109; Grimm Technologies Inc., Douglasville, GA, USA), which receives annual calibration by the manufacturer. The analyzer can detect a wide range of particle sizes, from 0.25 μm up to 32 μm [2,18]. The particles were classified by diameter (d) into six size categories: d < 0.5 μm, 0.5 ≤ d < 1.0 μm, 1.0 ≤ d < 3.0 μm, 3.0 ≤ d < 5.0 μm, 5.0 ≤ d < 10.0 μm, and d ≥ 10.0 μm; this categorization has been adopted in a previous study of bioaerosol characteristics associated with particulate matter (PM) [8]. The count and particle size measures were continuously recorded by the analyzer. To coordinate the bioaerosol sampling times and facilitate appropriate analysis, 5-min average values were used.

### 2.4. Statistical Analysis

A statistical analysis was carried out with IBM SPSS Statistics 24.0 (SPSS Inc., Chicago, IL, USA). Kolmogorov’s normality tests were performed to identify the utility of parametric or non-parametric tests. One-way ANOVAs with Duncan’s post hoc comparisons were used to compare the differences in the concentration of airborne microbes among the sampling sites. The relationship between particle number and bioaerosol concentration was examined using Pearson’s correlation coefficient.

In this study, the predictive model for bacterial and fungal bioaerosol concentration according to particle size was constructed by multiple linear regression (MLR). The predictive model with environmental variables can be presented as Equation (1):

(1) $$Y=\beta_{0}+\beta_{1}X_{1}+\beta_{2}X_{2}+\beta_{3}X_{3}+\cdots +\beta_{n}X_{n}+\varepsilon$$

where Y is a dependent variable (concentration of airborne bacteria and fungi), X_i_ denotes independent variables (indoor air parameters: size-segregated particle number concentration, temperature, and humidity), β_0_ denotes intercept coefficients, β_i_ denotes regression coefficients, and ε denotes random errors.

We performed stepwise MLR to construct the bioaerosol prediction model in each space. In order to evaluate the predictive ability of the regression model, each model was evaluated with the following methods: First, 90% of the samples (training set) were randomly selected to build the predictive model. Subsequently, the remaining 10% of the samples (test set) were used to validate the regression model. We also used standard statistical forecasting to obtain the mean absolute percentage error (MAPE), which explains the power of the MLR model for predicting the indoor bioaerosol concentration related to size-segregated particle number [11].

MAPE was calculated using Equation (2):

(2) $$MAPE=\;\overset{n}{\underset{t=1}{\sum }}\left|\frac{observed_{t\;}-predicted_{t}}{observed_{t}}\right|\;\times \;\frac{100\%}{n}$$

where *n* is the total sample size, *observed_t_* is the measured value of bioaerosols at time (*t*), and *predicted_t_* is the predicted value from the prediction model constructed using size-segregated particle number and meteorological conditions.

A MAPE value of less than 10% indicates excellent predictive accuracy, a value between 10 and 20% indicates good predictive ability, a value between 20 and 50% is acceptable accuracy, and a value over 50% indicates unacceptable predictive accuracy.

Finally, external validation was performed on the prediction model constructed at two medical facilities (GH-A and CL-A) using data from other hospitals with similar environmental conditions. GH-B and CL-B data were respectively used to validate the prediction models built using GH-A and CL-A. An analysis of the rate of agreement between the predicted values derived from each model and the measured values was done with the Bland–Altman method, representing mean bias and limit of agreement (LoA) [19]. The 95% limit of agreement was calculated as follows (3):

(3) $$\mathrm{Limit}\;\mathrm{of}\;\mathrm{agreement}\;(\mathrm{LoA})\;\Rightarrow \;[\bar{d}–1.96\mathrm{SD},\;\bar{d}+1.96\mathrm{SD}]$$

## 3. Results

### 3.1. Charaterization of Bacterial and Fungal Bioaerosols

**Figure 1:**
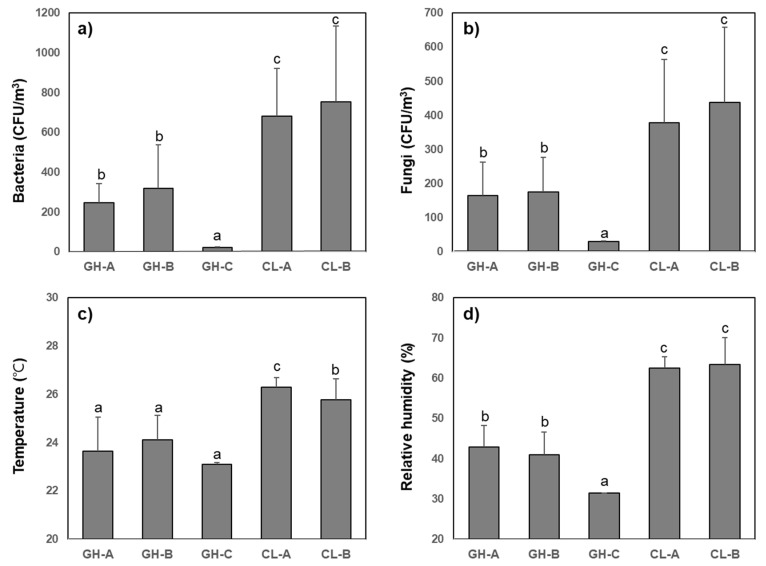
Distribution of airborne bacteria (**a**) and fungi (**b**), and meteorological conditions (**c,d**) in general hospitals (GH-A, GH-B, and GH-C) and clinics (CL-A and CL-B). ^a,b,c^ same letters indicate no significant difference based on Duncan’s multiple comparisons (*p* < 0.001).

The indoor bioaerosol concentrations and meteorological conditions measured in each hospital environment are shown in Figure 1. The average bacterial bioaerosol concentrations ranged widely, from 19 to 1534 CFU/m^3^. The highest concentration of airborne bacteria was 753 ± 382 CFU/m^3^ in CL-B, and 57% of the samples exceeded the indoor air quality guidelines for South Korea (800 CFU/m^3^). On the other hand, the lowest bacterial concentration (23 ± 3 CFU/m^3^) was found in GH-C, which had a HEPA filtration-equipped HVAC system meant to maintain a clean environment in the CSR.

The distribution of airborne fungi concentrations was similar to that of bacterial bioaerosols. The highest total mean fungal bioaerosol level was observed in CL-B (438 ± 220 CFU/m^3^) and the excess rate was 31% compared to the guidelines (500 CFU/m^3^). The fungal concentration in GH-C ranged from 30 to 32 CFU/m^3^, which was lower than the concentration at all other sampling sites. Both bacterial and fungal bioaerosol concentrations were significantly higher in clinics than in general hospitals (except for GH-C).

The ambient temperature ranged from 20.4 °C to 28.8 °C with an average of 24.4 °C and the relative humidity ranged from 30.2% to 69.3% with a mean level of 51.4%. There was no significant difference in temperature among general hospitals and the temperature was significantly higher in CL-B and CL-A. Relative humidity was the lowest in GH-C and significantly higher in CL-A and CL-B than in GH-A and GH-B.

### 3.2. Concentration and Distribution of Size-Segregated Particles

Table 2 presents the mean particle numbers in the six particle size categories. As with the bioaerosol distribution, size-segregated particle counts were at their lowest level in GH-C. The highest particle count was observed for PM_0.5_, with a particle size < 0.5 μm, at all investigated locations. Particles smaller than 0.5 μm were measured in a very wide range of 2,394,298–28,527,000 particle numbers/m^3^. Meanwhile, the average PM_0.5_ number exceeded the ISO 14644 recommended standard limit for ISO 8 cleanrooms (352,000 particles/m^3^, at all locations).

As particle size increased, particle number decreased, and the count of particles larger than 10 μm (super-coarse particles) was the lowest of all size categories investigated. Based on the particle size distribution, airborne particles mainly consisted of smaller particles in all investigated medical facilities.

### 3.3. Correlation between Bioaerosols and Size-Segregated Particle Number

**Figure 2:**
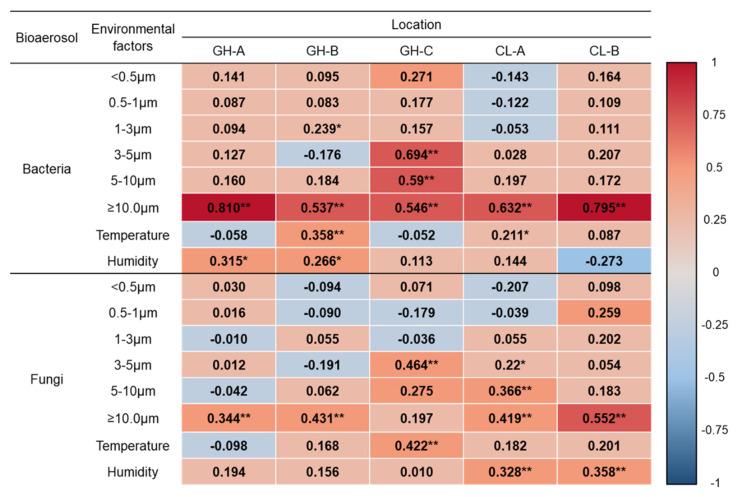
Pearson’s correlation coefficient matrix between bioaerosol levels and different particle sizes and meteorological conditions. * *p* < 0.05, ** *p* < 0.001.

The results of the correlation analysis conducted to identify the association between bioaerosol concentration and each particle size category are shown in Figure 2. Airborne bacteria and particle size showed a moderate to strong positive correlation with particle size > 10 μm at all sampling locations. In the case of GH-B, a weak positive correlation was observed for particle sizes of 1–3 μm, and in GH-C, a moderate to strong positive correlation was observed for coarse particles, such as particle sizes of 3–5 μm and 5–10 μm. Although the relationship was less strong than that seen for bacteria, the concentration of fungi was also weakly and moderately correlated with the concentration of particles larger than 10 µm. In GH-C, a moderate positive correlation was observed for particle sizes of 3–5 μm; and in CL-A, a weak positive correlation was observed for particles sizes of 5–10 μm.

The correlation analysis between bioaerosol levels and meteorological conditions (temperature and humidity) revealed a weak to moderate positive correlation. In the case of bacteria, a weak positive correlation was observed for both temperature and humidity in GH-B, and a weak positive correlation was observed for humidity in GH-A and temperature in CL-A. Airborne fungi and ambient temperature showed a moderate positive correlation in GH-C, while weak but significant positive correlations were observed between humidity and fungal bioaerosol in both clinics.

### 3.4. Prediction Models for Bacterial and Fungal Bioaerosols

A regression analysis was performed with the number concentrations of the six particle size categories and meteorological conditions as variables. A bioaerosol prediction model was constructed by stepwise MLR analysis, as shown in Table 3.

Particles > 10 μm significantly explained the bacterial bioaerosol concentration (prediction models for bacterial bioaerosols, PMB-1, *R^2^* = 0.634), and the explanatory power increased to 70.1% when humidity was additionally included as a variable in PMB-2. Based on Equation (2), the MAPE values of PMB-1 and PMB-2 were 40.3% and 38.9%, respectively, indicating an acceptable predictive accuracy (20–50%). PMB-5 (*R^2^* = 0.804), with a MAPE value of 8.5% (<10%), accurately predicted airborne bacterial bioaerosol concentration using the number of particles in the size categories of 1–3 μm, 3–5 μm, and 5–10 μm in GH-C.

On the other hand, in one of the clinics (CL-A), particles > 10 μm and temperature were selected for the prediction model. The MAPE value of PMB-7 (*R^2^* = 0.539) was 46.1%, indicating a reasonable ability to predict the level of airborne bacteria. 

In terms of fungal bioaerosols, the *R^2^* value of the regression model was lower than that of PMB. The two models constructed in GH-A (prediction models for fungal bioaerosols, PMF-1 and PMF-2) showed only 10.9–17.1% explanatory power, with MAPE values of 142.8% and 115.9% (>50%), respectively. This result may indicate that the selected models were inaccurate. For GH-C, the PMF-3 (*R^2^* = 0.197) and PMF-4 (*R^2^* = 0.293) models were proposed, but the MAPE value was 64.3–96.5%, indicating a low prediction accuracy. In CL-A, a total of four regression models were selected; the explanatory power of PMF-8 was the highest, at 48.9%, and the MAPE value was 42.5%. PMF-8 was the only model that could reasonably predict fungal bioaerosol concentration among PMFs. In PMF-8, super-coarse particles (PM > 10) and a subsection of coarse particles (PM3-4.9 and PM5-9.9) were selected as variables. In contrast to the other locations, humidity was additionally reflected in the prediction model.

When the regression models were constructed based on 90% of samples and tested with the remaining 10% of samples, the *R^2^* values between the training set and the test set were similar in all models.

### 3.5. External Validation of Selected Prediction Models

**Figure 3:**
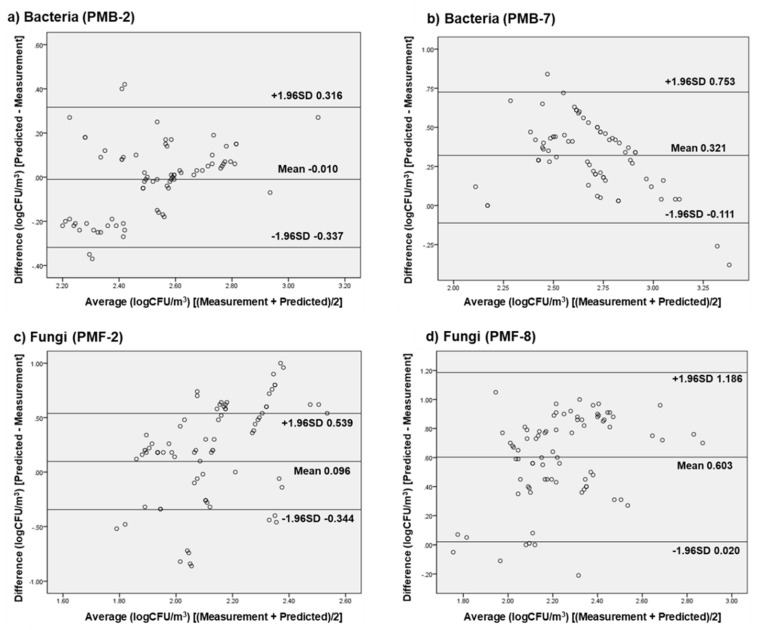
Comparison of biological aerosol concentrations between measured (actual) value and predicted value using the Bland–Altman plot. (**a,b**) Predictive models for bacterial bioaerosol and (**c,d**) predictive models for fungal bioaerosol.

PMB-2, PMB-7, PMF-2, and PMF-8 were finally selected for model verification in GH-A and CL-A. External validation was performed using data from hospitals that were similar in size to the hospital where the predictive model was constructed and with similar sources of pollution, HVAC systems, and concentrations of bioaerosols. The Bland–Altman results are shown in Figure 3. The *x*-axis represents the average of the measured values and the predicted values derived by the model, and the *y*-axis represents the difference between the predicted values and the measured values. The 95% confidence interval was expressed based on the mean difference ($\bar{d}$), and the acceptability of each predictive model was evaluated.

The LoA of PMB-2 was calculated as [−0.337, 0.316]. This model had acceptable forecasting ability except in the case of four-sample data, which were rarely out of the limit of agreement in the region of extremely low bacterial concentration. The lower and upper limits of the agreement of PMB-7 were 0.753 and −0.111, respectively. The selected prediction model was reliable except for the three-sample data, the $\bar{d}$ value of which was 0.321, indicating a tendency to overestimate at lower bacterial concentrations and occasionally produce unacceptable predictions in the highest concentration range.

Regarding fungal bioaerosols, a graphical analysis was also performed with PMF-2 for comparison purposes, although only PMF-8 was finally selected as the prediction model for airborne fungi. As expected, PMF-2 was an unacceptable model, consistent with the results of the MAPE analysis. PMF-8 was evaluated as reliable, except in the cases of six samples. PMF-8, with an LoA of [0.020, 1.186] and a $\bar{d}$ value of 0.603, tended to slightly overestimate fungal concentration.

As with the validation using the test set and the results of MAPE analysis, the prediction models for bacterial bioaerosols (PMB-2 and PMB-7) showed good predictive power in the external validation. Among the prediction models for airborne fungi, PMF-8 was evaluated as the only meaningful model for assessing airborne fungi levels.

## 4. Discussion

In this study, the predictability of bioaerosol (airborne bacteria and fungi) concentration was evaluated using size-segregated particle number and meteorological conditions in the hospital environment. We estimated the correlation between the particulate matter count according to particle size and bioaerosol levels and proposed a prediction model at each location. Furthermore, validation through the test set and calculation of MAPE were carried out to evaluate the predictive power of the model. Finally, external validation was performed to evaluate the usability of the model in other hospital environments using a Bland–Altman plot, a graphical comparison technique that is derived to evaluate the degree of agreement between the actual measured value and the expected value from the selected prediction models. For GH-C, where bioaerosol levels were measured in the CSR, external validation of the prediction models with data from other hospital environments was not performed because other hospitals would not allow measurements to be taken in the CSR.

Bacterial and fungal concentrations were distributed over a very wide range in the selected study areas. The average concentrations of bacteria and fungi were 371 and 253 CFU/m^3^, respectively, which were similar to values previously reported in Korea [20,21,22]. In GH-C, measurements were taken in the CSR, a department that sterilizes and stores medical supplies; thus, GH-C showed a significantly lower bioaerosol concentration compared with other hospital environments because the HEPA filters in the HVAC system effectively reduced the concentration of biological particles in the air [23]. Similarly, particle counts in all size categories were significantly lower in GH-C compared with the other sampling sites.

Several studies reported a correlation between bioaerosol levels and particulate matter concentration, but some conflicting studies found no correlation [11]. We presume that the inconsistencies arise from differences in the methods used to measure particulate matter. When particles are measured by mass concentration, they are classified broadly as PM1, PM2.5, and PM10. Therefore, the contribution of specific size of particles may be neglected or weakened because a correlation cannot be observed according to the as-subdivided categories. When particle size is divided with the size-segregated particle counting method, individual observation of correlation is more effective than mass concentration measurement, and correlations that were not observed by gravimetric methods may appear. A few studies have classified particles into various size ranges in the hospital environment [8]. Meanwhile, we were able to determine the association between bioaerosol levels and particulate matter concentration by analyzing the aerodynamic particle size distribution.

For bacteria, a strong positive correlation was observed for particle sizes > 10 μm in all facilities, and a moderate positive correlation was additionally observed for PM3-5 and PM5-10 in GH-C. A past study postulated that bacterial bioaerosols between 3 and 7.5 μm in size exist in an aggregated state, whereas bacteria that are 1–2 μm in size exist freely, and larger bacterial bioaerosols (>7.5 μm) are nasal- or oral-derived bacteria which tend to cluster and adhere to other large particles [24]. In this study, we found that airborne bacteria exist as larger aerosols combined with biological or non-biological particles rather than alone.

In previous studies, a moderate to strong positive correlation between airborne bacteria and size-segregated particle number was observed for particles ranging in size from 7.5 to 17.5 μm. On the other hand, a correlation was observed for particles in the range of 1–5 μm in the operating room and intensive care unit, but no correlation was observed for particles ≥ 5 μm in size because the size interval was too wide [2,8].

Regarding temperature, previous findings yielded inconsistent results [2,25], and humidity was found to either be uncorrelated or positively correlated with bacterial bioaerosol levels, which is in agreement with our results.

Interestingly, airborne fungi also showed a weak to moderate positive relationship with the concentration of particles > 10 μm in all sample spaces, but the relationship was not as strong as that between bacterial bioaerosol and particulate matter. Airborne fungi are typically single-celled fungal spores (2–5 μm in size) and multicellular fungal spores (>10 μm), which are dominant in indoor environments [26], indicating that the size distribution of fungal bioaerosols differs widely. Multicellular fungal spores may act as a source of contamination for larger fungal aerosols, as they are subsequently resuspended due to human activity [27]. Although large fungal spores can be removed by HVAC filtration under unoccupied conditions [28], larger fungal spores cannot be effectively removed from hospital wards occupied by humans (not only medical staff, but also patients and visiting guests) at investigated areas. In GH-C and CL-A, a weak to moderate, but significant, correlation was additionally observed at a particle size of 1–3 μm, suggesting that unicellular and multicellular fungal bioaerosols co-exist.

In some studies, no association was found between the concentrations of particulate matter and airborne fungi [29,30], but a correlation appeared by size-segregated particle counting similar to that observed our results [11]. Therefore, we emphasize that size subdivision of particles is essential when assessing bioaerosol concentration via particle number counts. Regarding meteorological conditions, there were no significant results except that temperature showed a moderate positive relationship in GH-C. Remarkably, a significant correlation was observed between humidity and size-resolved particle number only in clinics (CL-A and CL-B). Most species of fungi are difficult to grow in conditions where the relative humidity does not exceed 60% [31,32]. This may explain why no correlation with humidity was observed in general hospitals, where the relative humidity was in the range of 31.42–42.98%.

The correlation between airborne bacteria and fungi in indoor environments and the concentration of particulate matter > 10 μm in size was our most remarkable finding. Studies done in various indoor environments have shown that the biological particles generated during human occupancy are mostly large particles (>9 μm) [33,34]. However, in GH-C, the correlation between PM_3-5_ and PM_5-10_ and bioaerosol concentration was more pronounced than that of PM > 10, indicating that the contribution of larger particles derived from human skin, hair, and nasal and oral cavities was lower than that seen in other spaces. Since unauthorized personnel are not allowed in this location and medical personnel work to maintain the sterility of the environment, there are only a few sources of large biological particles, such as human shedding. Accordingly, most bacteria were found in the coarse particle category in the CSR.

Sources of indoor fungi include penetration from the outside as well as resuspension in indoor air [34]. Most indoor fungi are single-celled fungal spores, and most outdoor fungi are multicellular fungal spores [26]. GH-C, which was not affected by outside air, and unicellular fungal spores (particle size of 3–5 μm) showed a significant correlation with airborne fungi. In contrast, a significant correlation between fungal bioaerosols and the concentration of particles > 10 μm in size was observed because indoor airborne fungi were affected by the penetration of outdoor fungal spores, and their size gradually increased by combining with other particles in other hospital environments (GH-A, GH-B, CL-A, and CL-B) with natural ventilation.

Regarding the explanatory power of the prediction models, PMB was superior overall to PMF. PMB-2 (MAPE = 38.98%) and PMB-7 (MAPE = 46.1%) were similar to the MAPE (40.0–49.1%) of the bacterial prediction model proposed in previous studies [11]. Notably, PMB-5 showed very accurate predictive power (MAPE < 10%), suggesting that particle counting can reasonably be utilized as a surrogate measurement which can replace culture-based methods to monitor the levels of airborne bacteria in real-time.

The fungi prediction models were not suitable, except for PMF-8. In a related study that constructed a fungi prediction model using multiple linear regression, the MAPE value was 112.8–126.4%, which did not represent an acceptable fungal bioaerosol prediction model [11]. In this study, however, the forecasting accuracy (MAPE = 42.5%) was greatly improved by additionally considering the relative humidity, which may affect the distribution of indoor fungi, as well as the size-resolved particle counts. We proved that the limitations of the existing prediction models can be overcome by reflecting meteorological conditions in addition to size-segregated particle number. In this way, a more complete model could be proposed according to the environmental characteristics at each hospital environment.

External verification was subsequently performed to assess whether the selected prediction models could be utilized in other hospital environments. Three models (PMB-2, PMB-7, and PMF-8) showed excellent agreement on the Bland–Altman plot, reflecting a considerable improvement in the predictive power of the fungi model. Therefore, we concluded that the predictive model for bioaerosol concentration is quite useful in other, similar hospital environments.

Culture-based bioaerosol monitoring requires a skillful experimenter and laborious culture processes [35]. In addition, it is very difficult to measure bioaerosols in real-time with this method because the analytic process takes one to five days. A bioaerosol concentration reading is only valid at the time of measurement; therefore, a rapid microbial measurement method is required for public spaces used by a sensitive population that requires an immediate response to microbial contamination.

As a result, it is necessary to assess the concentration of biological particles in a hospital environment in real-time through surrogate factors such as size-segregated particle counts and meteorological conditions, as proposed in this study.

## 5. Conclusions

In this study, particulate matter was classified into six categories according to aerodynamic diameter, significant correlations were identified between bioaerosol concentration and particulate matter levels, and the selected variables were reflected in prediction models. Internal and external validation analyses were performed to determine the fit of the model. PMB showed an acceptable predictive ability for both general hospital and clinic sites, and PMF showed reasonable forecasting accuracy in a clinic environment with a relative humidity of 60% or more.

This study has several limitations. Since we did not perform a lot of sampling for each space in the hospital, we could not propose a model representing each ward. It is necessary to propose a bioaerosol prediction model that reflects the characteristics of a ward through long-term measurements. If environmental factors such as the number of occupants, outdoor PM, and ventilation frequency are added to the model, a more accurate model can be constructed, but the purpose of this study was to build a quick and simple bioaerosol prediction model through monitoring of real-time indoor particulate matter levels. In addition, this is the first study to perform external validation that obtained meaningful results by applying the prediction model to other similar environments.

There are several methods to detect specific species of bioaerosol in real-time [36,37,38], and in this study, the level of total culturable bioaerosol could be predicted. We suggest that a surrogate measurement is needed to detect pathogenic species and approach the level of bioaerosols. These surrogate evaluation methods are essential for hospital environments that require an immediate response to microbial contamination. Therefore, we suggest that additional research on the particle counting method is needed, especially in hospital spaces that require a pollution-free environment, such as operating rooms and intensive care units. The prediction models for bioaerosol would be more accurate and comprehensible through further research and validation in a larger number of hospitals.

By combining our bioaerosol prediction model with a periodic culture-based method, it is possible to more fully assess the microbiological quality of indoor air and contribute to solving problems related to the hospital environment, including HAIs.

## Figures and Tables

**Table 1:** Characteristics of the sampling sites selected for this study.

Type	Sampling Site	Sampling Point	No. of Samples	Potential Pollutant Source	Type of Cooling, Heating and Ventilation System
General hospital	GH-A	IM, SW, GW, TR, PR	240	Human activities	Central HVAC and natural ventilation
				(patients and medical staff) Outdoor	
	GH-B	IM, SW, GW, TR, PR	210	Human activities	
				(patients, visitors, and medical staff)	
				Outdoor	
	GH-C	CSR	135	Human activities (medical staff)	HEPA filtration in HVAC systems
Clinic	CL-A	TR, PR	215	Human activities	Natural ventilation
	CL-B	TR, PR	210	(patients and medical staff) Outdoor	

GH: general hospital, CL: clinic, IM: internal medicine, SW: surgical ward, GW: general ward, TR: treatment room, PR: patient room, CSR: central supply room, HEPA: High Efficiency Particulate Air Filter, HVAC: Heating, Ventilating and Air Conditioning System.

**Table 2:** Mean (SD) of particle numbers in the six size categories.

Location	Particulate Count/m^3^					
	<0.5 μm *	0.5–1 μm *	1–3 μm *	3–5 μm *	5–10 μm *	≥10.0 μm *
GH-A	16,403,812 ^c^	472,838 ^c^	46,582 ^d^	4997 ^d^	1685 ^d^	597 ^c,d^
	(6,035,471)	(407,205)	(43,969)	(3167)	(856)	(272)
GH-B	15,511,037 ^c^	273,434 ^b^	14,785 ^b^	1473 ^b^	878 ^b^	487 ^b^
	(11,136,194)	(271,467)	(7751)	(961)	(538)	(277)
GH-C	4,164,399 ^a^	89,704 ^a^	5718 ^a^	395 ^a^	141 ^a^	120 ^a^
	(781,951)	(10,169)	(1466)	(229)	(58)	(79)
CL-A	19,280,252 ^d^	549,781 ^c^	38,703 ^c^	3570 ^c^	1379 ^c^	527 ^b,c^
	(3,097,115)	(157,515)	(15,191)	(1687)	(532)	(161)
CL-B	12,510,489 ^b^	350,251 ^b^	32,733 ^c^	3001 ^c^	1328 ^c^	611 ^d^
	(4,256,087)	(162,571)	(10,625)	(1325)	(511)	(318)

GH: general hospital, CL: clinic. * ^a,b,c,d^ same letters indicate no significant difference based on Duncan’s multiple comparisons (*p* < 0.001).

**Table 3:** Prediction models for bacterial and fungal bioaerosols and evaluation of the predictive ability of each model. The *R^2^* value of the prediction model calculated through the test set and the forecasting accuracy (mean absolute percentage error (MAPE)) are additionally shown in this table.

Bioaerosol	Location	Regression Model	Training Set		Test Set		MAPE (%)
			*R^2^* (Adj *R^2^*)	*R* (*p*-Value)	*R^2^* (Adj R^2^)	*R* (*p*-Value)	
Bacteria	GH-A	PMB-1: logC_b_(CFU/m^3^) = (6.189 × 10^−4^) PM_>10_ + 1.971	0.644 (0.638)	0.802 (0.000)	0.625 (0.612)	0.791 (0.000)	40.3
		PMB-2: logC_b_(CFU/m^3^) = (6.093 × 10^−4^) PM_>10_ + 0.011H + 1.501	0.710 (0.701)	0.842 (0.000)	0.703 (0.695)	0.839 (0.000)	38.9
	GH-C	PMB-3: logC_b_(CFU/m^3^) = (6.358 × 10^−5^) PM_3-5_ + 1.336	0.482 (0.470)	0.694 (0.000)	0.455 (0.439)	0.675 (0.000)	53.1
		PMB-4: logC_b_(CFU/m^3^) = (6.977 × 10^−5^) PM_3-5_ + (1.691 × 10^−5^) PM_1-3_ + 1.236	0.739 (0.726)	0.859 (0.000)	0.741 (0.730)	0.861 (0.000)	26.0
		PMB-5: logC_b_(CFU/m^3^) = (5.713 × 10^−5^) PM_3-5_ + (1.613 × 10^−5^) PM_1-3_ + (9.555 × 10^−5^) PM_5-10_ + 1.232	0.817 (0.804)	0.904 (0.000)	0.853 (0.831)	0.924 (0.000)	8.5
	CL-A	PMB-6: logC_b_(CFU/m^3^) = (9.295 × 10^−4^) PM_>10_ + 2.026	0.535 (0.501)	0.732 (0.000)	0.583 (0.533)	0.764 (0.001)	61.2
		PMB-7: logC_b_(CFU/m^3^) = (1.015 × 10^−3^) PM_>10_ + 0.193 T - 3.086	0.564 (0.539)	0.751 (0.000)	0.590 (0.566)	0.768 (0.000)	46.1
Fungi	GH-A	PMF-1: logC_f_(CFU/m^3^) = (3.683 × 10^−4^) PM_>10_ + 1.917	0.122 (0.109)	0.349 (0.003)	0.116 (0.099)	0.341 (0.001)	142.8
		PMF-2: logC_f_(CFU/m^3^) = (3.545 × 10^−4^) PM_>10_ + 0.016H + 1.243	0.195 (0.171)	0.441 (0.001)	0.203 (0.185)	0.451 (0.003)	115.9
	GH-C	PMF-3: logC_f_(CFU/m^3^) = (3.742 × 10^−6^) PM_3-5_ + 1.496	0.216 (0.197)	0.464 (0.001)	0.225 (0.209)	0.475 (0.000)	96.5
		PMF-4: logC_f_(CFU/m^3^) = (3.161 × 10^−6^) PM_3-5_ + 0.018T + 1.131	0.325 (0.293)	0.570 (0.000)	0.301 (0.284)	0.549 (0.000)	64.3
	CL-A	PMF-5: logC_f_(CFU/m^3^) = (5.441 × 10^−4^) PM_>10_ + 2.240	0.176 (0.164)	0.419 (0.000)	0.231 (0.215)	0.481 (0.000)	101.8
		PMF-6: logC_f_(CFU/m^3^) = (5.619 X 10^−4^) PM_>10_ + 0.012H + 1.594	0.295 (0.275)	0.543 (0.000)	0.287 (0.264)	0.536 (0.001)	76.7
		PMF-7: logC_f_(CFU/m^3^) = (7.036 × 10^−4^) PM_>10_ + 0.007H + (3.302 × 10^−5^) PM_3-5_ + 1.398	0.460 (0.429)	0.678 (0.000)	0.417 (0.398)	0.646 (0.000)	58.2
		PMF-8: logC_f_(CFU/m^3^) = (6.338 × 10^−4^) PM_>10_ + 0.006H + (5.055 × 10^−5^) PM_3-5_ + (8.824 × 10^−5^) PM_5-10_ + 1.003	0.504 (0.489)	0.710 (0.000)	0.516 (0.496)	0.719 (0.000)	42.5

PMB: prediction models for bacterial bioaerosols, PMF: prediction models for fungal bioaerosols.
